# The administration of rtPA before mechanical thrombectomy in acute ischemic stroke patients is associated with a significant reduction of the retrieved clot area but it does not influence revascularization outcome

**DOI:** 10.1007/s11239-020-02279-1

**Published:** 2020-09-16

**Authors:** Rosanna Rossi, Seán Fitzgerald, Sara Molina, Oana Madalina Mereuta, Andrew Douglas, Abhay Pandit, Andreia M. Silva Santos, Blathnaid Murphy, Jack Alderson, Paul Brennan, Sarah Power, Alan O’Hare, Michael Gilvarry, Ray McCarthy, Klearchos Psychogios, Georgios Magoufis, Georgios Tsivgoulis, András Nagy, Ágnes Vadász, István Szikora, Katarina Jood, Petra Redfors, Annika Nordanstig, Erik Ceder, Niclas Dehlfors, Dennis Dunker, Turgut Tatlisumak, Alexandros Rentzos, John Thornton, Karen M. Doyle

**Affiliations:** 1 Department of Physiology and Galway Neuroscience Centre, School of Medicine, National University of Ireland, Galway, University Road, Galway, Ireland; 2 CÚRAM–Centre for Research in Medical Devices, National University of Ireland Galway, Galway, Ireland; 3 Centro Universitário Unievangélica, Anápolis, Goiás Brasil; 4 Department of Radiology, Royal College of Surgeons in Ireland, Beaumont Hospital, Dublin, Ireland; 5 Cerenovus, Galway, Ireland; 6 Stroke Unit, Metropolitan Hospital, Piraeus, Greece; 7 Department of Neuroradiology, Metropolitan Hospital, Piraeus, Greece; 8 Department of Neurointerventions, National Institute of Clinical Neurosciences, Budapest, Hungary; 9 Department of Neurology, Sahlgrenska University Hospital, Gothenburg, Sweden; 10 Department of Clinical Neuroscience, Institute of Neuroscience and Physiology, Sahlgrenska Academy at University of Gothenburg, Gothenburg, Sweden; 11 Department of Interventional and Diagnostic Neuroradiology, Sahlgrenska University Hospital, Institute of Clinical Sciences, University of Gothenburg, Gothenburg, Sweden

**Keywords:** Thrombolysis, thrombectomy, Acute ischaemic stroke, Blood clot

## Abstract

Both intravenous thrombolysis (IVT) and mechanical thrombectomy (MT) are evidence-based treatments for acute ischemic stroke (AIS) in selected cases. Recanalization may occur following IVT without the necessity of further interventions or requiring a subsequent MT procedure. IVT prior to MT (bridging-therapy) may be associated with benefits or hazards. We studied the retrieved clot area and degree of recanalization in patients undergoing MT or bridging-therapy for whom it was possible to collect thrombus material. We collected mechanically extracted thrombi from 550 AIS patients from four International stroke centers. Patients were grouped according to the administration (or not) of IVT before thrombectomy and the mechanical thrombectomy approach used. We assessed the number of passes for clot removal and the mTICI (modified Treatment In Cerebral Ischemia) score to define revascularization outcome. Gross photos of each clot were taken and the clot area was measured with ImageJ software. The non-parametric Kruskal–Wallis test was used for statistical analysis. 255 patients (46.4%) were treated with bridging-therapy while 295 (53.6%) underwent MT alone. By analysing retrieved clot area, we found that clots from patients treated with bridging-therapy were significantly smaller compared to those from patients that underwent MT alone (H_1_ = 10.155 p = 0.001*). There was no difference between bridging-therapy and MT alone in terms of number of passes or final mTICI score. Bridging-therapy was associated with significantly smaller retrieved clot area compared to MT alone but it did not influence revascularization outcome.

## Highlights

- Thrombi from 550 AIS patients have been grouped according to the administration (or not) of intravenous thrombolysis before thrombectomy and the mechanichal thrombectomy approach used.
- Clot area, number of passes and final recanalization outcome have been analysed.
- Bridging-therapy was associated with significantly smaller retrieved clot area compared to mechanical thrombectomy alone.
- No differences in terms of number of passes or final recanalization outcome were found between brigding-therapy and mechanical thrombectomy alone groups.

## Introduction

Several clinical trials have demonstrated that mechanical thrombectomy (MT) is an effective therapy for acute ischemic stroke (AIS) compared to the use of intravenous thrombolysis (IVT) only [1–5]. However, whether pre-treatment with intravenous thrombolytics (bridging-therapy) significantly affects MT success is a matter of debate. Earlier studies reported conflicting results [1–8], even though more recent studies seem to conclude that MT alone may offer comparable safety and efficacy to bridging-therapy [9–14]. Ongoing randomized trials are continuing to address this question.

We hypothesised that IVT may reduce retrieved clot area and may influence revascularisation outcome compared to MT alone. This multi-center international study investigated the influence of bridging-therapy compared to MT alone on the retrieved clot area and on revascularization outcome in a cohort of 550 AIS patients treated with aspiration, stentriever or rescue-therapy.

## Materials and methods

### Patient cohort

The study here presented is a multi-centre prospective study involving four stroke centres in Europe: Beaumont Hospital (Dublin, Ireland), Sahlgrenska University Hospital (Gothenburg, Sweden), National Institute of Clinical Neurosciences (Budapest, Hungary) and Metropolitan Hospital, (Piraeus, Greece). This study was conducted in accordance with the ethical standards of the Declaration of Helsinki and its amendments [15], by approval of the regional hospital ethics committees and National University of Ireland Galway research ethics committees (16-SEPT-08). We included only patients > 18 years, having been treated with mechanical thrombectomy for AIS and whose thrombus material was available to be analyzed. For each patient an anonymized data abstraction form was collected, which contained pertinent procedural data, including occlusion location, type of device used for MT, number of passes for clot removal and final modified Treatment In Cerebral Ischemia (mTICI) score [16].

### Study plan

**Fig. 1:**
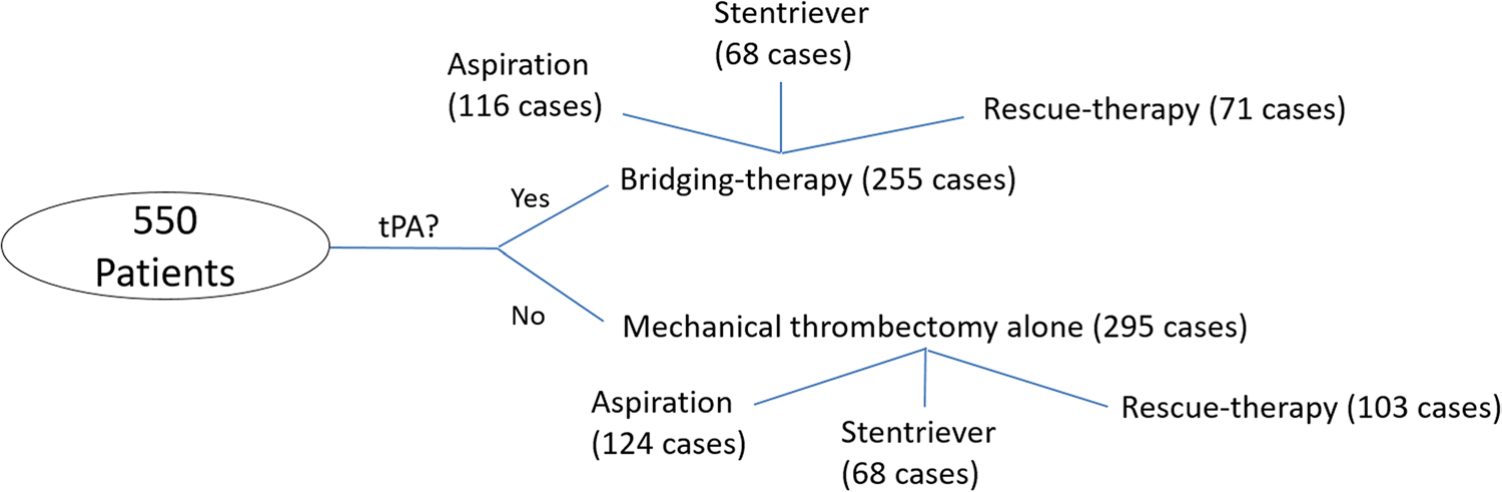
Study plan

This study included patients that underwent endovascular treatment with or without previous administration of rtPA between March 2017 and March 2019 for acute occlusion of a large artery in the anterior or posterior circulation. The study plan is illustrated in Fig. 1. Treatment approach was at the discretion of the clinician. Clots from 550 AIS patients were collected separately for each procedural pass by the four stroke centres and shipped in formalin 10% to NUI Galway, where a gross photo of each was taken with a Canon EOS 1300D Camera. Clot area was then measured with ImageJ software (NIH, Bethesda, Maryland, USA). To measure the area of a fragment, the gross photo was opened with ImageJ, the scale was set and the Polygon tool was used to draw a region of interest around a fragment of the clot. For cases having multiple passes the retrieved clot area of each pass was measured and then summed to give the retrieved clot area of the whole case. At least two certified radiologists assessed recanalization in post-treatment angiograms, using the mTICI grading scale [16].

Cases were first divided into two groups according to the administration (or not) of rtPA before the endovascular treatment (bridging-therapy vs mechanical thrombectomy alone). Then, cases were further classified into three sub-groups according to the overall approach used for MT (i.e. aspiration, stentriever and rescue-therapy). Patients treated with MT alone included cases ineligible for rtPA administration, due to time-window exclusion and/or other contraindications. In accordance with European and International Guidelines on stroke management [7–19], the inclusion criteria for rtPA administration are: (i) diagnosis of ischemic stroke causing measurable neurological deficit; (ii) treatment within 4.5 h from onset. Exclusion criteria for rtPA administration include current haemorrhage or conditions that increase risk of haemorrhage. Additional exclusion criteria for rtPA administration between 3 and 4.5 h from onset are: (i) age > 80 years, (ii) severe stroke (NIHSS > 25); (iii) history of diabetes and prior stroke; (iv) taking an oral anticoagulant. Suspected stroke etiology was reported according to the TOAST classification system [20].

Patients in rescue-therapy subgroups included all the cases when the first line approach failed and a switch to a different device or technique was required.

### Statistical analysis

IBM SPSS-25 software was used for statistical analysis. Kolmogorov–Smirnov test and Shapiro–Wilk test indicated that quantitative variables did not follow a standard normal distribution. Therefore, the non-parametric Kruskal–Wallis test was used to assess statistically significant difference among the groups, with a level of statistical significance set at p < 0.05 (two-sided). Results are reported as median [IQ1-IQ3] or number and % of cases.

## Results

### Baseline characteristics of the patients

Among the 550 cases considered, 255 patients (46.4%) were treated with bridging-therapy while 295 (53.6%) were treated with mechanical thrombectomy alone; baseline clinical characteristics of both groups of patients are reported in Table 1. There were no significant differences between the groups observed.

**Table 1:** Baseline clinical characteristics of the two groups of patients, bridging-therapy and mechanical thrombectomy alone

Pre-thrombectomy conditions	Bridging-therapy cases (N = 255)	Mechanical thrombectomy alone cases (N = 295)	Statistical analysis
Patients with cardioembolic suspected etiology	95(37.3%)	111(37.6%)	N = 550 H_1_ = 1.003 p = 0.317
Patients with large artery atherosclerosis suspected etiology	58(22.7%)	58(19.7%)	
Patients with other suspected etiology^a^	15(5.9%)	15(5.1%)	
Patients with cryptogenic/unknown suspected etiology	87(34.1%)	111 (37.6%)	
Admission NHISS score	17[12–21]^b^	16[11–20]^c^	N = 550 H_1_ = 0.552 p = 0.457
Post-thrombectomy complications			
Symptomatic and asymptomatic haemorrhage	10(11.4%)	24(19.0%)	N = 214^d^ H_1_ = 2.279 p = 0.131

^a^Other suspected etiology included: arterial dissection, pulmonary embolism, hypercoagulable states, or hematologic disorders

^b^Three patients were unconscious/intubated

^c^Ten patients were unconscious/intubated

^d^Data available only for a subgroup of patients, 88 receiving tPA and 126 treated with MT alone, for a total of 214 patients. Data given as N(%) of cases or median [IQ1, IQ3]

### Bridging-therapy and occlusion location

Occluded vessels for the two groups of patients are reported in Table 2. In almost half of cases (46.7%), the occluded vessel was the M1 segment of middle cerebral artery (MCA). The occlusion of one or more segments/branches of MCA represented the majority of occluded vessels in this study (58.1%), followed by ICA (15.6%) and tandem and dual occlusions (14.0%). This proportion was maintained when the cases where split in bridging-therapy vs MT alone, with similar distribution (Table 2).

**Table 2:** Occluded vessels in the whole cohort of patients and in the two groups, bridging-therapy and mechanical thrombectomy alone

Occluded vessel(s)	All the cases (N = 550)	Bridging-therapy cases (N = 255)	Mechanical thrombectomy alone cases (N = 295)
MCA,M1	257(46.7%)	122(47.8%)	135(45.8%)
MCA,M2	50(9.1%)	25(9.8%)	25(8.5%)
MCA,M3	3(0.5%)	2(0.8%)	1(0.3%)
Vertebro/basilar	42(7.6%)	17(6.7%)	25(8.5%)
ICA	86(15.6%)	36(14.1%)	50(16.9%)
PCA	8(1.5%)	1(0.4%)	7(2.4%)
ACA	1(0.2%)	1(0.4%)	0(0.0%)
CCA	2(0.4%)	1(0.4%)	1(0.3%)
Tandem and dual occlusions	77(14.0%)	34(13.3%)	43(14.6%)
MCA, multiple segments/branches	10(1.8%)	9(3.5%)	1(0.3%)
3 or more occlusion location	14(2.5%)	7(2.7%)	7(2.4%)

Data given as N(%) of cases

*MCA* middle cerebral artery, *ICA* internal carotid artery, *PCA* posterior cerebral artery, *ACA* anterior cerebral artery, *CCA* common carotid artery

### Bridging-therapy is associated with a smaller area of retrieved clots

By measuring retrieved clot area, we observed a median of 0.33 [0.16–0.59] cm^2^ for clots retrieved from patients treated with bridging-therapy, while the median for clots retrieved from patients treated with MT alone was 0.39 [0.22–0.82] cm^2^. Bridging-therapy is associated with the retrieval of significantly smaller clots (H_1_ = 10.155, p = 0.001*).

### Bridging-therapy does not improve revascularization outcome compared to mechanical thrombectomy alone in patients with successful retrieval of clot material

We analysed the influence of IVT administration before mechanical thrombectomy on the total number of passes for the three different approaches and the results are shown in Table 3. There was no statistically significant difference between the two groups, whether rtPA had been administered or not, irrespective of the approach considered (H_1_ = 0.488, p = 0.485 for aspiration procedures, H_1_ = 0.903,p = 0.342 for stentriever procedures, H_1_ = 1.276,p = 0.259 in case of rescue-therapy). Aspiration and stentriever procedures both required a median of 1 [1, 2] passes to remove the clot, while with rescue-therapy, a median of 4 [2–5] and 4 [3–5] passes were needed, respectively for bridging-therapy and MT alone.

**Table 3:** Impact of bridging-therapy vs mechanical thrombectomy alone on total number of passes

Variable	Overall technique	Bridging-therapy	Mechanical thrombectomy alone	Statistical analysis
Number of passes	Aspiration	1 [1, 2]	1 [1, 2]	N = 240, H_1_ = 0.488, p = 0.485
	Stentriever	1 [1, 2]	1 [1, 2]	N = 136, H_1_ = 0.903, p = 0.342
	Rescue-therapy	4 [2–5]	4 [3–5]	N = 174, H_1_ = 1.276, p = 0.259

Data given as median[IQ1-IQ3]

Similarly, the administration of rtPA before mechanical thrombectomy did not influence significantly the final mTICI score regardless of the approach used during the endovascular treatment (aspiration, stentriever or rescue-therapy, Table 4). Final recanalization was similar in procedures that used aspiration or stentrievers (Table 4). Considering mTICI 2c-3 as a good revascularization outcome, satisfactory outcomes were obtained for at least 63% of cases in aspiration and stentriever groups, but when rescue-therapy was needed the percentage of successful revascularization lowered to 52% (Table 4).

**Table 4:** Impact of bridging-therapy vs mechanical thrombectomy alone on final mTICI score

Variable	Overall technique					
	Aspiration		Stentriever		Rescue-therapy	
Final mTICI Score	Bridging-therapy (N = 116)	Mechanical thrombectomy alone (N = 124)	Bridging-therapy (N = 68)	Mechanical thrombectomy alone (N = 68)	Bridging-therapy (N = 71)	Mechanical thrombectomy alone (N = 103)
0	0(0.0%)	2(1.6%)	2(2.9%)	1(1.5%)	1(1.4%)	2(1.9%)
1	3(2.6%)	0(0.0%)	0(0.0%)	0(0.0%)	3(4.2%)	2(1.9%)
2a	3(2.6%)	2(1.6%)	4(5.9%)	3(4.4%)	10(14.1%)	8(7.8%)
2b	23(19.8%)	32(25.8%)	19(27.9%)	13(19.1%)	20(28.2%)	37(35.9%)
2c	23(19.8%)	24(19.4%)	13(19.1%)	10(14.7%)	15(21.1%)	23(22.3%)
3	64(55.2%)	64(51.6%)	30(44.1%)	41(60.3%)	22(31.0%)	31(30.1%)
Statistical analysis	N = 240, H_1_ = 0.316, p = 0.574		N = 136, H_1_ = 3.440, p = 0.064		N = 174, H_1_ = 0.156, p = 0.693	

Data given as N(%) of cases

## Discussion

In the treatment of AIS, time is brain. It is crucial to act as soon as possible in order to reduce the damage to the brain following a lack of blood supply. The impact of bridging-therapy on final revascularization outcome, and on the overall procedural outcome, is still a matter of debate. However, despite an increased risk of intracranial haemorrhage [21], in the absence of contraindications to rtPA, the standard therapy for large vessel occlusion (LVO) above six NIHSS [22] is a combination of MT and rtPA (the so-called “bridging-therapy”) for all eligible patients [17–19, 21, 23, 24].

We hypothesised that prior IVT may be associated with smaller retrieved clots and may affect the rates of recanalization as measured by mTICI and affect the number of passes required to remove clot. Our results demonstrate that prior IVT did indeed reduce clot size, but did not influence pass number or recanalization outcome.

Tissue plasminogen activator promotes the breakdown of fibrin polymers; therefore, in theory, the reduced clot size in patients undergoing bridging-therapy should be expected, even though, to the best of our knowledge, this is the first study that shows the association of bridging-therapy to smaller retrieved clots. It must be acknowledged that we can only conclude on the effect of IVT in the cases where thrombectomy resulted in extraction of at least part of the clot. We do not have data in the cases of patients who did not undergo thrombectomy due to successful thrombolysis and or clinical improvement, or had unsuccessful thrombectomy, with no clot extracted. We also acknowledge that the two patient populations in this study, i.e. those undergoing respectively bridging-therapy or MT alone are different from each other. The population treated with MT alone included patients ineligible for rtPA for several reasons, from time window exclusion and ongoing anticoagulation for atrial fibrillation to other major contraindications [17–19, 23, 24]. Moreover, the MT alone group also included some patients that did not receive rtPA because they had suffered a too severe stroke (NIHSS > 25). However, we can confirm that parameters such as NIHSS score on admission, suspected etiology and occlusion location were similar between the groups in this study. Therefore, it seems reasonable to attribute the smaller retrieved clot area of the bridging-therapy population to the effect of prior IVT treatment.

The number of passes to remove clot may depend on several factors such as clot burden, clot composition, stability, and porosity as well as operator experience [25]. The impact of each of these factors has not been fully elucidated yet. However, it is clear that a lower number of procedural passes correlates with a better outcome [25].

We found bridging-therapy and MT alone equivalent in terms of revascularization outcome, with similar number of passes and final mTICI scores for aspiration and stentriever procedures. As expected, rescue-therapy cases were associated with more passes and worse final recanalization, but bridging-therapy and MT alone were also equivalent in rescue-therapy cases. Our finding of the absence of any particular difference between bridging-therapy and MT alone on both number of passes and recanalization rates, is in line with several studies previously published [26, 27]. As previously acknowledged, in this study we could not include the patients who recanalised with IVT only, without need for further endovascular treatment. Nonetheless, on the basis of our findings comparing MT alone to bridging-therapy, the results show that IVT makes the clot smaller, perhaps causing it to break into pieces, diminishing in this way its size, and perhaps increasing the risk of distal embolism to smaller arteries [28]. IVT may soften the clot, however, since number of passes and final degree of recanalization were similar, rtPA does not seem to have a significant facilitating effect on detachment of clot during MT.

A main advantage of rtPA is that the treatment is readily available to centres that do not have the advanced technology needed or interventionalists with specialized skill sets or in remote areas that cannot provide an in-house stroke specialist 24/7 [29]. It is still unclear if, for patients with suspected AIS whether rtPA versus immediate mechanical thrombectomy should be prioritized [30] but the previous administration of rtPA may certainly be of help for patients living in areas where the primary referral hospital is a local stroke centre with no availability of mechanical thrombectomy option. We observed no significant difference in incidence of procedural related haemorrhage in the bridging-therapy and MT alone groups. Although bridging-therapy did not result in better revascularization outcome compared to MT alone, it is also true that it did not result in worse revascularization outcome than MT alone, at least considering the two groups compared in this study. Furthermore, a benefit of pre-treatment with IVT may be that rtPA still circulating may be helpful to dissolve the remnants of the thrombus. A clinical trial is currently investigating the possible benefits of local intra-arterial treatment post MT for this reason [31]. It is possible that the lack of significant effect on number of passes to remove clot and final revascularisation outcome that we observed in this study reflects the fact that IVT may have both beneficial and detrimental effects due to its mechanism of action, but on balance does not adversely affect the outcome in MT procedures.

### Study limitations and strengths

One of the limitations of this study is the lack of complete data about patients undergoing bridging-therapy. In particular, we have not probed information about the number of “drip and ship” patients or full details about IVT administration, timings, dosage and kind of drugs used, although this should be studied in the future. Inclusion of more extensive post procedural outcome data, such as lesion volume of infarct, mortality rate, 90 day mRS scores would also benefit future work. Another limitation is that we measured an area rather than a volume to give an indication of the size of the extracted clot, although we believe the area measurements are representative of the extracted clot burden.

However, a main strength of this study is the large patient population arising from four dedicated stroke centres in Europe, which reduces the effect of possible differences in clinical approach across the hospitals, giving robustness to our findings.

## Conclusion

The present study highlighted that the administration of rtPA before mechanical thrombectomy, in patients who did not recanalise with IVT only and where thrombectomy resulted in extraction of at least a part of the clot, was associated with a significant reduction of retrieved clot area, although it did not impact the number of passes or final mTICI score. Future studies will be performed to investigate also other important parameters, like the sex and the age of the patients as well as the time from groin puncture to recanalization, which may differ between bridging-therapy and MT alone patients, influencing then the final outcome.

## References

[1] Berkhemer OA, Fransen PS, Beumer D, Investigators MRCLEAN (2015). A randomized trial of intra-arterial treatment for acute ischemic stroke. N Engl J Med.

[2] Campbell BC, Mitchell PJ, Kleinig TJ (2015). EXTEND-IA investigators endovascular therapy for ischemic stroke with perfusion-imaging selection. N Engl J Med.

[3] Goyal M, Demchuk AM, Menon BK (2015). ESCAPE trial investigators randomized assessment of rapid endovascular treatment of ischemic stroke. N Engl J Med.

[4] Saver JL, Goyal M, Bonafe A (2015). SWIFT PRIME investigators stent-retriever thrombectomy after intravenous t-PA vs. t-PA alone in stroke. N Engl J Med.

[5] Jovin TG, Chamorro A, Cobo E (2015). REVASCAT trial investigators thrombectomy within 8 hours after symptom onset in ischemic stroke. N Engl J Med.

[6] Bracard S, Ducrocq X, Mas JL (2016). THRACE investigators mechanical thrombectomy after intravenous alteplase versus alteplase alone after stroke (THRACE): a randomised controlled trial. Lancet Neurol.

[7] Muir KW, Ford GA, Messow CM (2017). PISTE investigators endovascular therapy for acute ischaemic stroke: the pragmatic ischaemic stroke thrombectomy evaluation (PISTE) randomised, controlled trial. J NeurolNeurosurg Psychiatry.

[8] Mocco J, Zaidat OO, von Kummer R (2016). THERAPY trial investigators aspiration thrombectomy after intravenous alteplase versus intravenous alteplase alone. Stroke.

[9] Weber R, Nordmeyer H, Hadisurya J (2017). Comparison of outcome and interventional complication rate in patients with acute stroke treated with mechanical thrombectomy with and without bridging thrombolysis. J Neurointerv Surg.

[10] Coutinho JM, Liebeskind DS, Slater LA (2017). Combined intravenous thrombolysis and thrombectomy vs thrombectomy alone for acute ischemic stroke: a pooled analysis of the SWIFT and STAR studies. JAMA Neurol.

[11] Abilleira S, Ribera A, Cardona P (2017). Outcomes after direct thrombectomy or combined intravenous and endovascular treatment are not different. Stroke.

[12] Rai AT, Boo S, Buseman C (2018). Intravenous thrombolysis before endovascular therapy for large vessel strokes can lead to significantly higher hospital costs without improving outcomes. J Neurointerv Surg.

[13] Leker RR, Pikis S, Gomori JM, Cohen JE (2015). Is bridging necessary? A pilot study of bridging versus primary stentriever-based endovascular reperfusion in large anterior circulation strokes. J Stroke Cerebrovasc Dis.

[14] Alonso de Leciñana M, Martínez-Sánchez P, García-Pastor A (2017). Mechanical thrombectomy in patients with medical contraindications for intravenous thrombolysis: a prospective observational study. J Neurointerv Surg.

[15] WMA (2013). World Medical Association Declaration of Helsinki: ethical principles for medical research involving human subjects. JAMA.

[16] Goyal M, Fargen KM, Turk AS (2014). 2C or not 2C: defining an improved revascularization grading scale and the need for standardization of angiography outcomes in stroke trials. J Neurointerv Surg.

[17] The American Heart Association and American Stroke Association (2018). Released updated ischaemic stroke guidelines that were published in Stroke, and released during the International Stroke Conference 2018 (ISC; 24–26 January 2018.

[18] Ferro JM (2017). European stroke organization guideline for the diagnosis and treatment of cerebral venous thrombosis – endorsed by the European Academy of Neurology. Eur J Neurol.

[19] Turc G, Bhogal P (2019). European stroke organisation (ESO)–european society for minimally invasive neurological therapy (ESMINT) guidelines on mechanical thrombectomy in acute ischaemic stroke endorsed by stroke alliance for Europe (SAFE). Eur Stroke J.

[20] Adams HP, Bendixen BH, Kappelle LJ (1993). Classification of subtype of acute ischemic stroke. definitions for use in a multicenter clinical trial. TOAST. Trial of Org 10172 in acute stroke treatment. Stroke.

[21] Emberson J, Lees KR, Lyden P (2014). Effect of treatment delay, age, and stroke severity on the effects of intravenous thrombolysis with alteplase for acute ischaemic stroke: a meta-analysis of individual patient data from randomised trials. Lancet.

[22] Meyer BC, Lyden PD, Faha F (2009). The modified national institutes of health stroke scale (mNIHSS): its time has come. Int J Stroke.

[23] Albers GW, Marks MP, Kemp S (2018). Thrombectomy for stroke at 6 to 16 hours with selection by perfusion Imaging. N Engl J Med.

[24] Nogueira RG, Jadhav AP, Haussen DC (2018). Thrombectomy 6 to 24 hours after stroke with a mismatch between deficit and infarct. N Engl J Med.

[25] Kharouba R, Gavriliuc P, Yaghmour NE (2019). Number of stentriever passes and outcome after thrombectomy in stroke. J Neurorad.

[26] Goyal M, Menon BK, van Zwam WH (2016). Endovascular thrombectomy after large-vessel ischaemic stroke: a meta-analysis of individual patient data from five randomised trials. Lancet.

[27] Phan K, Dmytriw AA, Maingard J (2017). Endovascular thrombectomy alone versus combined with intravenous Thrombolysis. World Neurosurg.

[28] Kaesmacher J, Boeckh-Behrens T, Simon S (2017). Risk of thrombus fragmentation during endovascular stroke treatment. Am J Neuroradiol.

[29] Hess DC, Audebert HJ (2013). The history and future of telestroke. Nat Rev Neurol.

[30] Perez De La Ossa N, Abilleira S, Ribo M (2018). Drip and ship versus direct ship: the RACECAT study. Endovascular Today.

[31] Renú A, Blasco J, Millán M (2019). The chemical optimization of cerebral embolectomy trial: Study protocol. Int J Stroke.

